# Supplementary material for: Embryonic Stage of Congenital Zika Virus Infection Determines Fetal and Postnatal Outcomes in Mice
Source: Viruses. 2021 Sep 11;13(9):1807. doi: 10.3390/v13091807 (PMC8473443; doi:10.3390/v13091807)
Supplement: Supplementary file 1 [file viruses-13-01807-s001.zip › viruses-1342267-supplementary.pdf]

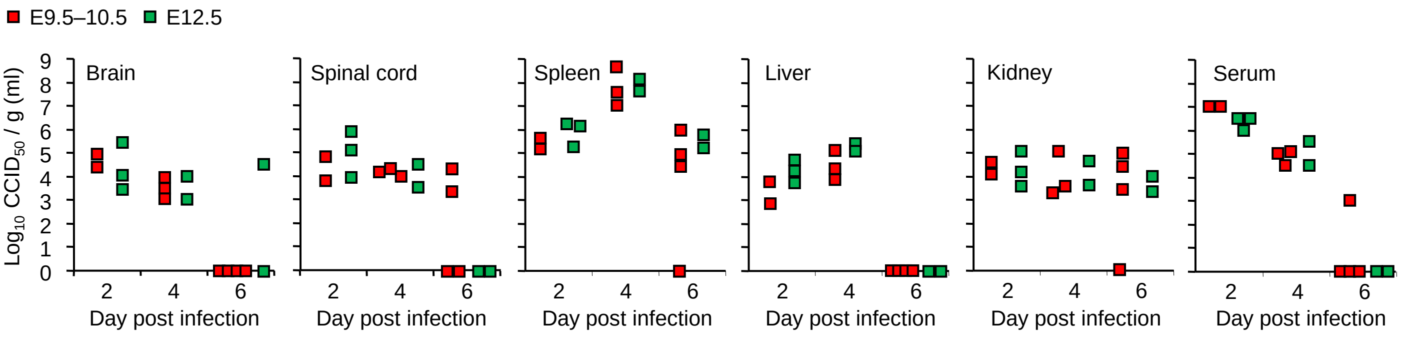

Supplemental Figure S1. Virus titers in maternal tissues. Pregnant IFNα/β receptor knockout (IFNAR$^{-/-}$) mice were infected with Zika virus (ZIKV) PRVABC59 at embryonic day 9.5 (E9.5)–E10.5 (red square) or E12.5 (green square) and their tissues harvested 2, 4, or 6 days post infection (dpi). Viral titers were determined using the 50% cell culture infective dose ($CCID_{50}$) assay. Statistical analysis was performed using two-way ANOVA.

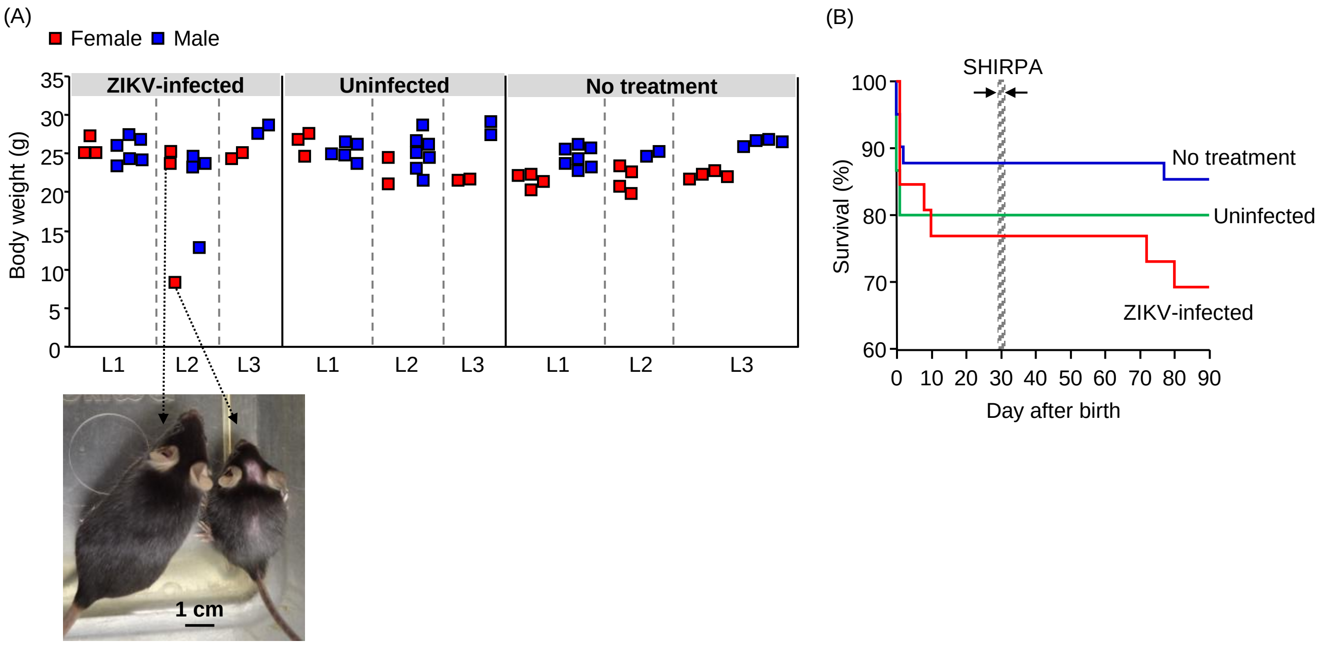

Supplemental Figure S2. Offspring infected at E12.5 in adulthood. (A) Reduced weight of infected offspring at 10 weeks after birth. Dams were infected subcutaneously (s.c.) with PRVABC59 at E12.5 (ZIKV-infected), inoculated s.c. with 2MEM at E12.5 (uninfected), or not treated throughout pregnancy and the postnatal period (no treatment). Offspring weight was measured 10 weeks after birth. Individual litters are indicated on the x-axis; each square represents a single offspring. Vertical dashed grey lines separate each litter. Dorsal view of smaller female offspring compared with that of female littermate. (B) Survival over 90 days after birth. SHIRPA was performed on offspring at 29–31 days of age (4-week-old), as indicated by the shaded area. The log-rank test was used for the statistical analysis of survival rates.

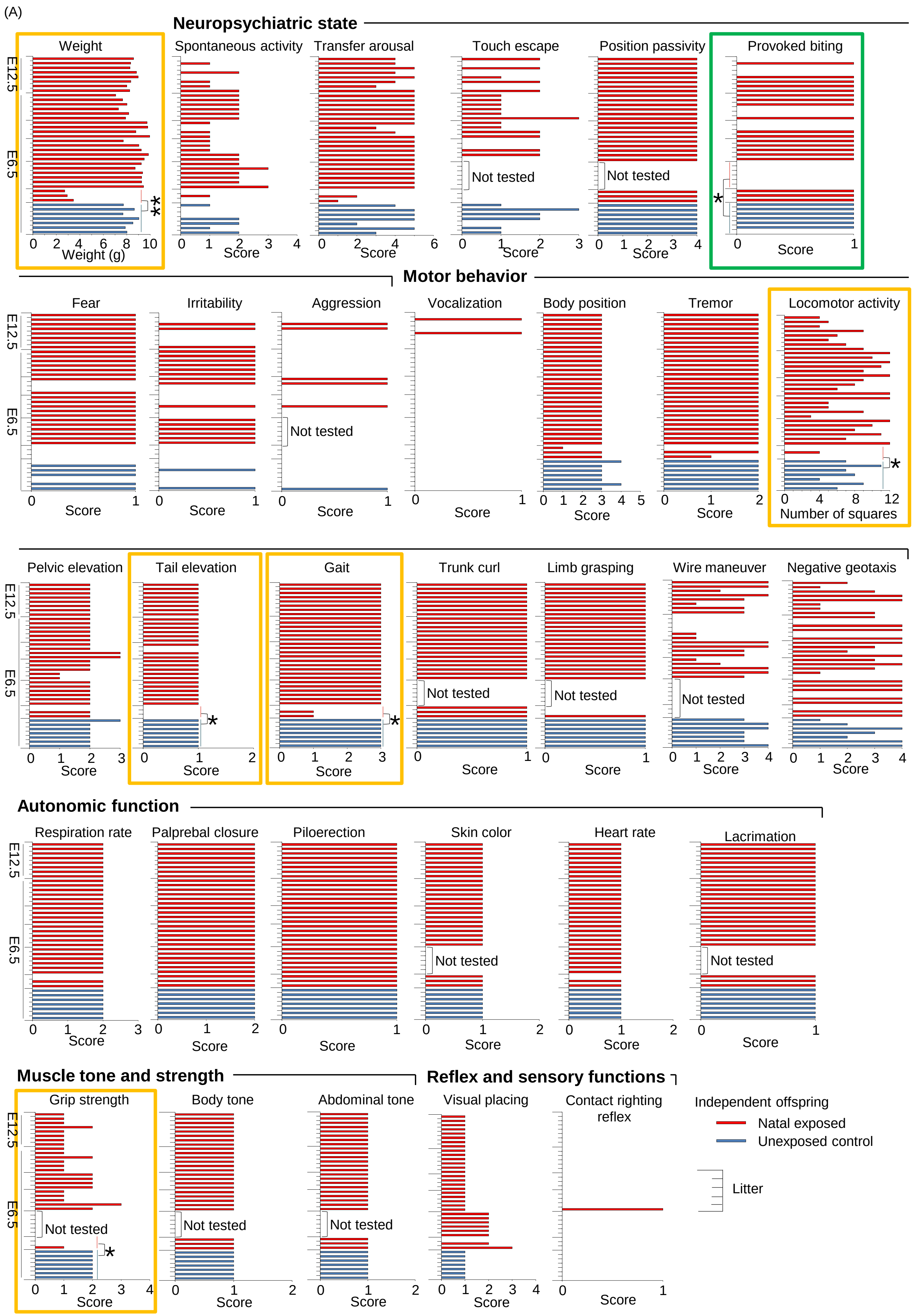

(A)
Neuropsychiatric state
Weight
Weight (g)
Spontaneous activity
Transfer arousal
Touch escape
Position passivity
Provoked biting
Not tested
Score
E12.5
E6.5
Motor behavior
Fear
Irritability
Aggression
Vocalization
Body position
Tremor
Locomotor activity
Number of squares
Pelvic elevation
Tail elevation
Gait
Trunk curl
Limb grasping
Wire maneuver
Negative geotaxis
Autonomic function
Respiration rate
Palpebral closure
Piloerection
Skin color
Heart rate
Lacrimation
Muscle tone and strength
Grip strength
Body tone
Abdominal tone
Reflex and sensory functions
Visual placing
Contact righting reflex
Independent offspring
Natal exposed
Unexposed control
Litter

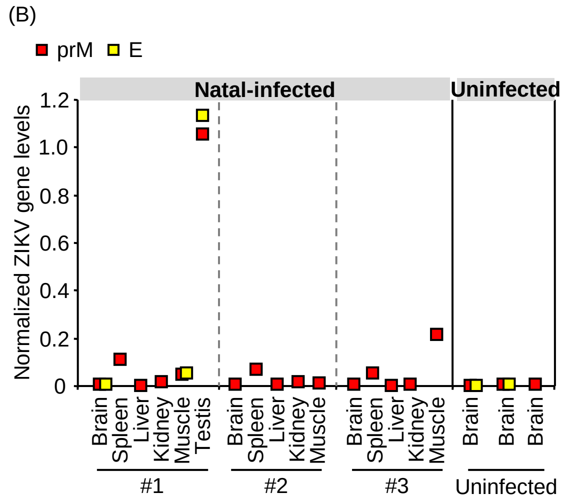

Supplemental Figure S3. Offspring outcomes after ZIKV Natal RGN infection. (A) SHIRPA results for offspring born to dams Natal RGN-infected (n = 6 litters) or uninfected (n = 1 litter) at 3 weeks post-birth. Pregnant IFNAR$^{-/-}$ dams crossed with IFNAR$^{-/-}$ males were infected subcutaneously (s.c.) with 1 × 10^4 $CCID_{50}$ of Natal RGN at E6.5 or E12.5, as previously described [26, 41, 42]. IFNAR$^{-/-}$ mice on a C57BL/6J background were provided by P. Hertzog (Monash University, Melbourne, VIC, Australia) [77]. Each histogram represents a single SHIPRA protocol test. Each bar represents a single offspring with red bars indicating mice born to Natal RGN-infected dams and blue bars indicating mice born to dams inoculated with RPMI1640 (Thermo Fisher Scientific, Inc., Waltham, MA, USA) containing 2% FBS. Longer lines along the y-axis separate the litters. Yellow boxes are used to highlight tests where the observably abnormal offspring performed significantly different from the offspring born to uninfected dams. The green box represents where other mice born to Natal RGN-infected dams performed significantly different to the mice born to uninfected dams. Some offspring did not undergo certain tests, as indicated by the “not tested” label. **, $p < 0.01$; *, $p < 0.05$. *T*-test with unequal variance or Kolmogorov-Smirnov test was used for statistical analysis. (B) ZIKV RNA levels in the offspring tissues. ZIKV RNA levels in the indicated tissues of offspring born to Natal RGN-infected dams at E6.5 determined by qRT-PCR with ZIKV prM or E gene-specific primers. ZIKV RNA levels were normalized to RPL13 mRNA levels. The vertical dashed gray lines represent each offspring. Three brains from uninfected mice were used to determine the detection limit.

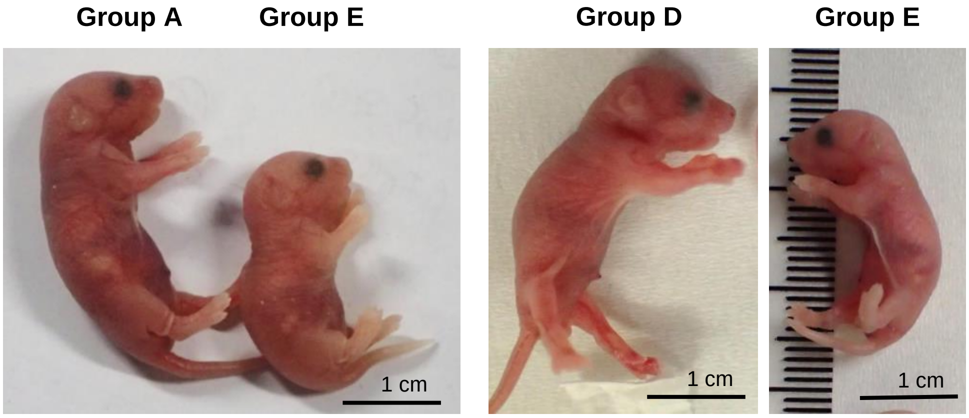

Supplemental Figure S4. Size of one-day-old offspring. Dams in Groups A and D had neutralizing antibodies before ZIKV challenge at E9.5, as shown in Figure 4A. Dams in Group E did not have neutralizing antibodies before ZIKV challenge at E9.5. The size of one-day-old offspring in Groups A and D was visually bigger than that in Group E. Representative offspring from each group are shown.

Supplemental Table S1. Mouse pregnancy models.

Dam	Sire	Inoculation dose	Virus strain	Route of infection	Infection	Inspection	Offspring outcomes	Ref
ICR	ICR	6.5×10^3 PFU	SZ01	Cerebroventricular space/LV of feral brain	E13.5	E16.5/18.5	Microcephaly, fetal brain infection	[33]
ICR	ICR	6.5×10^3 PFU	VEN/2016	LV of feral brain	E13.5	E18.5	Microcephaly, fetal brain infection	[34]
Swiss	Swiss	$5–10 \times 10^2$ PFU	Senegal_2015	intraplacental	E10.5	E18.5	Fetal resorption, fetal death	[73]
			Philippines_2012	intraplacental	E10.5	E18.5	Reduced fetal head weight	
			Thailand_2014	intraplacental	E10.5	E18.5	Reduced fetal head/brain weight	
C57BL/6	C57BL/6	3.4×10^5 , 1×10^5, 3.4×10^4 PFU	PRVABC59	Retrobulbar	E9.5	E17.5	Fetal demise, fetal resorption, IUGR, neonatal death	[64]
C57BL/6	C57BL/6	1.5×10^5 PFU	FSS13025	IVAG	E4.5	E18.5	Fetal growth defect, fetal brain infection	[28]
					E8.5	E18.5	No fetal growth defect but fetal brain infection	
$Irf3^{-/-}Ifr7^{-/-}$	$Irf3^{-/-}Ifr7^{-/-}$	1.5×10^5 PFU	FSS13025	IVAG	E4.5	E18.5	Reduced fetal weight, fetal body infection	
					E8.5	E18.5	No fetal growth defect but fetal body infection	
$IFNAR^{-/-}$	C57BL/6	1.5×10^5 PFU	FSS13025	IVAG	E4.5	E12.5	Fetal resorption	
					E8.5	E18.5	Reduced fetal weight, fetal body infection	
$IFNAR^{-/-}$	C57BL/6	10^3 PFU	H/PF/2013	SC	E6.5/E7.5	E13.5,E15.5	Spontaneous abortion, fetal demise, IUGR, fetal head infection	[29]
C57BL/6 + α-ifnar1-Ab	C57BL/6	10^3 PFU	H/PF/2013	SC	E6.5/E7.5	E13.5,E15.5	IUGR, fetal head infection	
$IFNAR^{-/-}$	C57BL/6	10^3 PFU	PRVABC59	SC	E7.5	E14.5	Fetal resorption, mild IUGR, fetal infection	[37]
			DAKAR41524	SC	E7.5	E14.5	Fetal resorption, fetal infection	
$IFNAR^{-/-}$	$IFNAR^{-/-}$	$10^{3, 4, 5, 6}$ $CCID_{50}$	Natal RGN	SC	E6.5	E17.5/E18.5	IUGR, deformed fetal/placental mass, abortion, fetal head infection	[26]
					E12.5	E17.5/E18.5	Deformed fetal/placental mass, abortion, fetal head infection	
$IFNAR^{-/-}$	$IFNAR^{-/-}$	10^4 $CCID_{50}$	P6-740	SC	E12.5	E17.5	Deformed fetal/placental mass, fetal head infection,	[41]
C57BL/6	C57BL/6	$\tilde{}3.4 \times 10^2$ $TCID_{50}$	MEX1-44	LV of fetal brain	E14.5	E18.5	Fetal brain infection	[32]
						P5	Reduced fetal body size, reduced fetal brain size and weight, Neonatal death by P10	
		$\tilde{}3.4 \times 10^2$ $TCID_{50}$	MR766	LV of fetal brain	E14.5	E18.5	Reduced brain size	
						P0	Neonatal death at P0/P1, reduced brain size	
C57BL/6	C57BL/6	1.7×10^3 $TCID_{50}$	MEX1-44	LV of fetal brain	E14.5	E17.5	Fetal brain infection	[31]
						P3	Microcephaly, growth restriction of offspring	
C57BL/6	C57BL/6	4.5×10^2 PFU	SZ01	IP	E13.5	E17.5/P1	Fetal brain infection, fetal brain development↓	[36]

C57BL/6	BALB/c	2.5×10^7 or $15 \text{ x } 10^5$ $TCID_{50}$	FB-GWUH-2016	Retrobulbar	E3.5	E17.5	Fetal loss, reduced fetal weight	[27]
					E12.5	E17.5	No outcome	
					E3.5	adulthood	Reduced survival, learning and memory impairments in male offspring	
SJL	SJL	1×10^3, 4×10^{10}, 1×10^{12} PFU	Brazilian clinical isolate	IV	E10.5–13.5	P0	IUGR, microcephaly, ocular abnormality, offspring brain infection	[74]
C57BL/6	C57BL/6	1×10^3, 4×10^{10}, 1×10^{12} PFU	Brazilian clinical isolate	IV	E10.5–13.5	P0	No outcome, no offspring infection	
C57BL/6	C57BL/6	5×10^2 PFU	SZ01	IA	E15.5	P0/P8	Offspring brain infection and reduced brain size	[35]
						P40	Visual and motor deficits, reduced body weight and brain volume	
C57BL/6	C57BL/6	10^4 PFU	PRVABC59	IP	E8.5	P0–P120	No apparent defects at birth but growth impediment and neurobehavioral deficits (reduced locomotor and cognitive deficits)	[75]
Swiss-Webster + α-ifnar1-Ab	Swiss-Webster	10^4 PFU	HN16	SC	E4.5	1–6 weeks after birth	Small head size but normal brain weight of female offspring	[30]
					E8.5		Small head size but normal brain weight of male/female offspring, growth restriction of male offspring	
					E12.5		No outcome	
AG129	AG129	1×10^2 $CCID_{50}$	P6-740	SC	E7.5	E17.5, E19.5	Fetal brain infection, IUGR	[76]
						P0–P45	Reduced body weight and head length, higher mortality by P35	
						8–16 weeks after birth	Healing loss	

Ref., reference; LV, lateral ventricle; E, embryonic day; IUGR, intrauterine growth restriction; IVAG, intravaginal; SC, subcutaneous; Ab, antibody; P, days after birth; IP, intraperitoneal; IV, intravenous; IA, intraamniotic.
